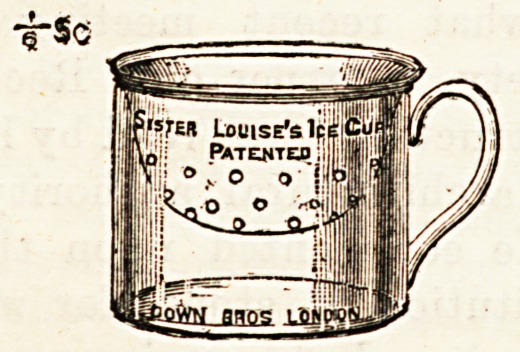# The "Sister Louise" Ice Cup

**Published:** 1895-09-21

**Authors:** 


### PRACTICAL DEPARTMENTS.

### THE "SISTER LOUISE" ICE CUP.

"Sister Louise" must be congratulated upon a very useful invention, and one which, so far as we know, is the only effort yet made to supply what is certainly a sick-room "want." Though a saucer and basin answer every practical requirement, it is not the tidiest or most elegant arrangement for the reception of ice, and our illustration shows how extremely neat and pretty is the substitute which has been patented by its inventor under the above name. We quite fell in love with it at Messrs. Down's the other day, and prophesy for it an ever-increasing popularity when once it is known. The little apparatus consists just of a deep glass cup with a handle, into which fits a saucer of the same material perforated in the centre for the drainage of moisture. It was a very happy thought which prompted the use of coloured glass, and the shaded red and blue tints chosen make the little cup a very dainty ornament, in addition to its qualities of use and convenience. For hospital use it would, of course, be made in earthenware, or some more durable substance. It is to be obtained from the well-known surgical instrument makers, Messrs. Down Bros., St. Thomas's Street, S.E., familiar to all visitors to Guy's Hospital.

## Figures and Tables

**Figure f1:**